# Differential changes in end organ immune cells and inflammation in salt-sensitive hypertension: effects of increasing M2 macrophages

**DOI:** 10.1042/CS20240699

**Published:** 2024-07-16

**Authors:** Shobana Navaneethabalakrishnan, Bethany L. Goodlett, Hannah L. Smith, Robert A. Montalvo, Alyssa Cardenas, Brett M. Mitchell

**Affiliations:** Department of Medical Physiology, Texas A&M University College of Medicine, Bryan, TX, U.S.A.

**Keywords:** hypertension, kidney, lymphatics, macrophages, ovaries, testes

## Abstract

Salt-sensitive hypertension (SSHTN) is associated with M1 macrophage polarization and inflammatory responses, leading to inflammation-associated lymphangiogenesis and functional impairment across multiple organs, including kidneys and gonads. However, it remains unclear whether promoting M2 macrophage polarization can alleviate the hypertension, inflammation, and end organ damage in mice with salt sensitive hypertension (SSHTN). Male and female mice were made hypertensive by administering nitro-L-arginine methyl ester hydrochloride (L-NAME; 0.5 mg/ml) for 2 weeks in the drinking water, followed by a 2-week interval without any treatments, and a subsequent high salt diet for 3 weeks (SSHTN). AVE0991 (AVE) was intraperitoneally administered concurrently with the high salt diet. Control mice were provided standard diet and tap water. AVE treatment significantly attenuated BP and inflammation in mice with SSHTN. Notably, AVE promoted M2 macrophage polarization, decreased pro-inflammatory immune cell populations, and improved function in renal and gonadal tissues of mice with SSHTN. Additionally, AVE decreased lymphangiogenesis in the kidneys and testes of male SSHTN mice and the ovaries of female SSHTN mice. These findings highlight the effectiveness of AVE in mitigating SSHTN-induced elevated BP, inflammation, and end organ damage by promoting M2 macrophage polarization and suppressing pro-inflammatory immune responses. Targeting macrophage polarization emerges as a promising therapeutic approach for alleviating inflammation and organ damage in SSHTN. Further studies are warranted to elucidate the precise mechanisms underlying AVE-mediated effects and to assess its clinical potential in managing SSHTN.

## Introduction

Hypertension (HTN) is a pervasive cardiovascular disorder that poses a significant global health challenge, as elevated blood pressure (BP) is a key risk factor for cardiovascular events, such as stroke and myocardial infarction, and renal complications [1–4]. This widespread health concern demonstrates notable diversity in its origins and clinical manifestations. Among the diverse phenotypes of HTN, salt sensitive HTN (SSHTN) stands out as a distinctive and intriguing subset that is characterized by BP fluctuations in response to variations in salt intake [5,6]. In the United States, about half of adults are diagnosed with HTN, with approximately 50% demonstrating SSHTN [7–9]. Salt sensitivity is a multifaceted phenomenon linked to various physiological, environmental, genetic, and demographic elements [10,11]. Noteworthy demographic indicators of salt sensitivity include gender, ethnicity, and age [10,12]. Research suggests that post-menopausal women, African American communities, and elderly individuals are at increased susceptibility to salt sensitivity [13,14]. However, the intricate biological underpinnings of salt sensitivity remain incompletely understood.

Over years of research, substantial evidence has accumulated that highlights the significance of chronic, low-grade inflammation in the development and progression of HTN, particularly in individuals who exhibit sensitivity to dietary salt intake [15–17]. In hypertensive individuals, inflammation can lead to endothelial dysfunction, arterial stiffness, and vascular remodeling, all of which contribute to elevated BP [18]. In turn, HTN can exacerbate inflammation by triggering oxidative stress and the release of pro-inflammatory cytokines [19]. This bidirectional relationship between HTN and inflammation underscores the importance of addressing both factors in the prevention and management of cardiovascular diseases.

Studies in both human and animal models have provided compelling evidence linking systemic inflammation to the development and exacerbation of SSHTN [15]. The inflammatory response to hypertensive stimuli involves the activation of both innate and adaptive immune pathways [20]. Macrophages represent a predominant immune cell population involved in SSHTN-associated inflammatory responses [15,21]. Due to their plasticity, macrophages can transition between pro-inflammatory M1 and anti-inflammatory M2 phenotypes in response to external signals [22]. High salt intake has been demonstrated to skew macrophage polarization toward a pro-inflammatory phenotype, thereby promoting vascular inflammation and endothelial dysfunction [23,24]. Research utilizing chemical and genetic modification of macrophages has revealed that the elimination of M1 macrophages enhances endothelial function and reduces systemic inflammation, highlighting the pivotal role of M1 macrophages in HTN development [25–27]. Studies conducted by our laboratory and others have demonstrated that SSHTN is associated with increased M1 macrophage polarization, inflammation, and inflammation-associated lymphangiogenesis in various organs, including kidneys [28–30]. Our recent findings also revealed an increase in M1 macrophages, along with a concomitant decrease in M2 macrophages, within the gonads of mice with SSHTN. This shift was associated with inflammation and an increase in lymphatic density in both testes and ovaries [31]. Nonetheless, it remains largely uncertain how macrophage polarization impacts BP, inflammation, and end organ damage in mice with SSHTN. Therefore, we hypothesized that increasing M2 macrophages would decrease BP and alter immune cell populations and mitigate the inflammation in kidneys and gonads of mice with SSHTN. We further hypothesized that these changes would be associated with decreased lymphatic density and improved overall function in the kidneys, testes, and ovaries.

The renin–angiotensin system (RAS) plays a critical role in maintaining cardiovascular homeostasis and is implicated in the development of HTN and cardiovascular disorders [32]. Angiotensin II (Ang II), an octapeptide derived from the enzymatic conversion of the decapeptide Angiotensin I (Ang I) by angiotensin-converting enzyme (ACE), serves as the principal effector molecule within the RAS. Ang II exerts its biological effects through two distinct receptor subtypes: AT1R (angiotensin type-1 receptor) primarily involved in vasoconstriction, inflammation, growth, and fibrosis, and AT2R (angiotensin type-2 receptor), which serves to counterbalance the actions mediated by AT1R [32,33]. Another counteractive mechanism is facilitated by Ang- [1–7], a heptapeptide, produced by the enzymatic deactivation of Ang II by ACE-2 [34,35]. In addition to directly inhibiting Ang II, Ang- [1–7] elicits a range of beneficial effects, including vasodilation, anti-inflammatory actions, and inhibition of cellular proliferation by activating the G protein–coupled receptor Mas [36,37]. AVE0991 (AVE), a Mas receptor agonist, has emerged as a promising therapeutic candidate due to its potential for vasodilation, as well as its anti-inflammatory and anti-fibrotic properties [1–7,38–40]. AVE has been shown to promote a shift in macrophage polarization toward the M2 phenotype through selective interactions with the Mas receptor [39]. In the present study, we investigated the effectiveness of AVE in reducing BP, changing immune cell populations, and decreasing inflammation in renal and gonadal tissues by modulating macrophage polarization. SSHTN was induced in male and female mice, some of which received intraperitoneal injections of AVE each day during the 3-week high salt diet phase. Here, we report the effects of M2 macrophage induction on renal and gonadal inflammation, lymphatics, and function in SSHTN mice.

## Methods

### Animal models

Experimental procedures involving animals were conducted with prior approval from the Texas A&M University Institutional Animal Care and Use Committee (IACUC: 2022-0083) and were performed in accordance with the guidelines outlined in the NIH Guide for the Care and Use of Laboratory Animals. Wild-type C57BL/6J mice were procured from Jackson Laboratories (Bar Harbor, ME) at 8–10 weeks of age and acclimatized for a period of 2 weeks. To induce SSHTN, male and female mice were administered nitro-L-arginine methyl ester hydrochloride (L-NAME; 0.5 mg/ml; Sigma, St. Louis, MO) in drinking water for 2 weeks, followed by a 2-week washout period. Subsequently, the mice were subjected to a 4% salt diet (Teklad Envigo, Huntingdon, U.K.) for 3 weeks (SSHTN) [29,41]. At the onset of the diet phase, a group of SSHTN mice was randomly assigned to the treatment group and received AVE injections (0.58 μmol per kg/body weight/day; Sigma) intraperitoneally each day (SSHTN+AVE) [39]. Mice in the control group were maintained on a standard diet and tap water for 7 weeks. All water and diets were provided *ad libitum*. Each group in the study comprised 14 male and female mice, out of which 6 mice were designated for flow cytometry, while the remaining were used for other biochemical assays (PCR, sperm parameters, and immunofluorescence). Mice were killed by exsanguination under 5% inhaled isoflurane anesthesia, with death confirmed by cervical dislocation, at the end of the 7-week model.

### Flow cytometry

Kidneys, testes, and ovaries were prepared for flow cytometric analysis as described in our companion paper, ‘Differential Changes in End Organ Immune Cells and Inflammation in Salt-Sensitive Hypertension: Effects of Lowering Blood Pressure’. Data were acquired using a Cytek Aurora 5L flow cytometer with SpectroFlo software v3.2.0 (Cytek Biosciences, Fremont, CA). Populations of up to 500,000 cells were analyzed using FlowJo v10.8 (FlowJo, LLC, Ashland, OR). Results are expressed as percentages of respective parent populations. Gating strategies for all samples were justified by referencing unstained specimens and compensation controls and are the same as shown in the companion paper. Antibody details are provided in Online Table I.

Methodology for all other experiments, gating strategies, PCR primer sequences, and statistical analyses are the same as described in our companion paper entitled ‘Differential Changes in End Organ Immune Cells and Inflammation in Salt-Sensitive Hypertension: Effects of Lowering Blood Pressure,’ as well as previous studies from our laboratory [29,31,42–44].

### Study approval

All animal procedures performed in mice were approved by the Texas A&M University IACUC in accordance with the *NIH Guide for the Care and Use of Laboratory Animals*.

## Results

### AVE treatment decreased systolic BP and improved renal immune cells and inflammation in male SSHTN mice

**Figure 1:**
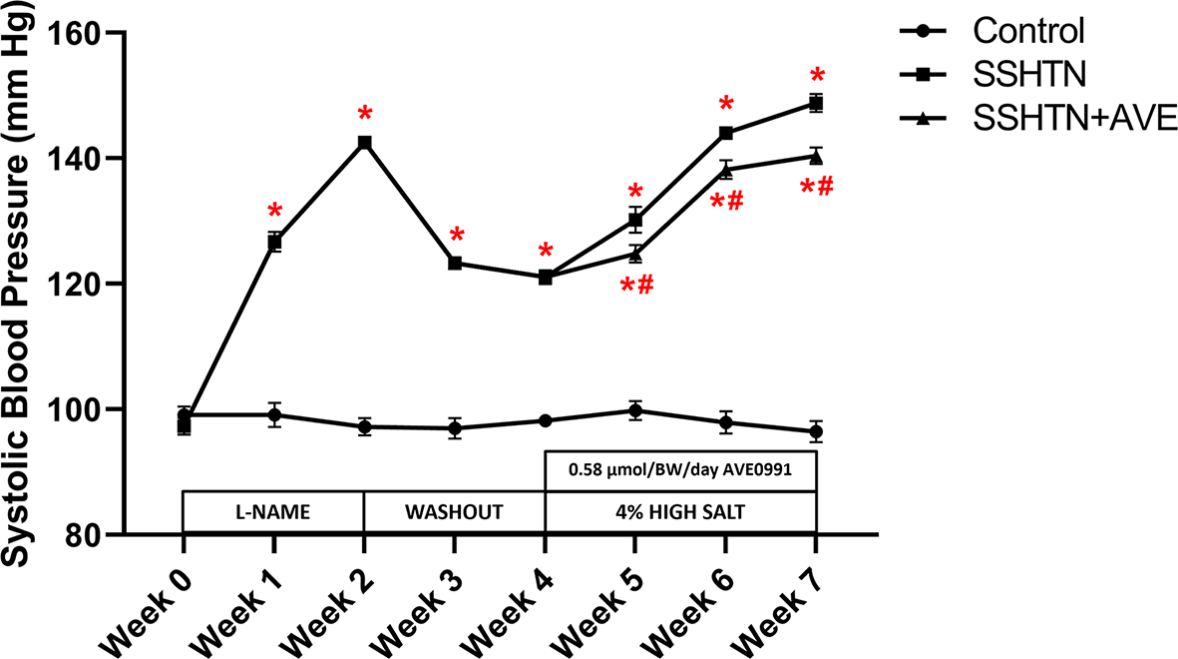
AVE0991 treatment attenuated SBP in male mice with SSHTN SBP measurements in untreated control, SSHTN, and AVE0991 (AVE)-treated SSHTN male mice. Blood pressures were taken weekly via tail cuff. Results are presented as mean ± SEM and statistical analyses were performed with a one-way ANOVA (*n* = 5 per group). *P<0.05 vs control mice and #*P*<0.05 vs SSHTN 0mice.

Increased renal and gonadal M1 macrophages have been observed in mice with SSHTN [30,31]. However, it is not known whether this altered macrophage polarization causes inflammation and end organ damage in the kidneys and gonads of mice with SSHTN. To evaluate whether manipulating macrophage polarization toward an M2 phenotype can reduce BP and inflammation, AVE was administered via daily intraperitoneal injection to SSHTN mice concurrently with a high salt diet for the final 3 weeks of the study. Consistent with previous data from our laboratory, a significant increase in systolic BP (SBP) was observed in SSHTN mice compared with controls each week (Figure 1). SSHTN+AVE mice had a significantly decreased SBP compared with SSHTN mice each week of the high salt diet phase, although SBP levels remained significantly higher than those of the control group throughout (Figure 1).

**Figure 2:**
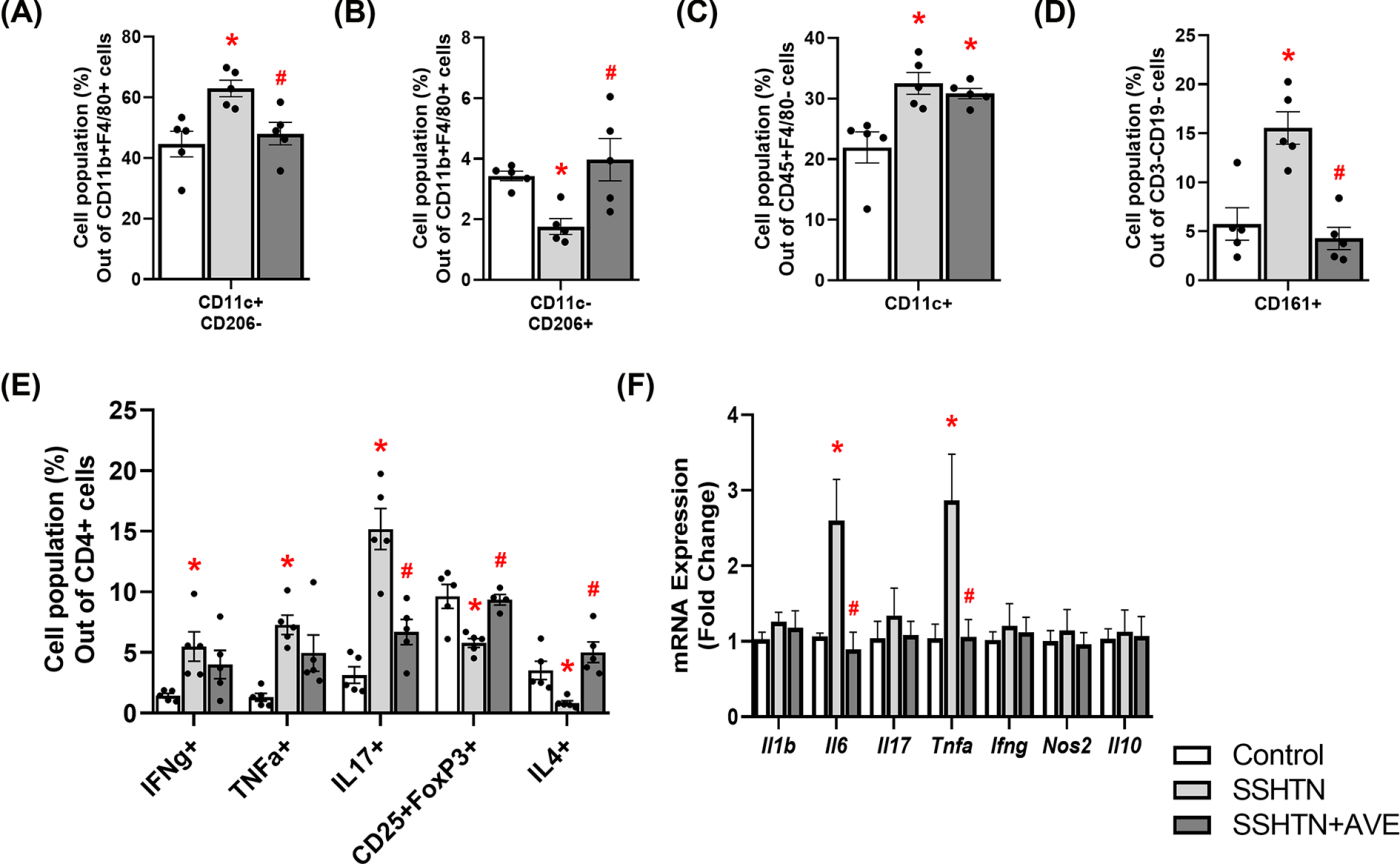
AVE0991 treatment differentially altered immune cells and inflammation in the kidneys of male mice with SSHTN Flow cytometric data examining renal populations of (**A**) M1 macrophages, (**B**) M2 macrophages, (**C**) DCs, (**D**) NK cells, and (**E**) T cells in control, SSHTN, and SSHTN+AVE male mice. Each cell population is shown as a percentage of its respective parent population. (**F**) Renal cytokine expression changes in control, SSHTN, and SSHTN+AVE male mice. Results are presented as mean ± SEM and statistical analyses were performed with one-way ANOVA (*n* = 4–6 per group). *P<0.05 vs control mice and #*P*<0.05 vs SSHTN mice.

Flow cytometric analysis unveiled a significant increase in pro-inflammatory M1 macrophages (Figure 2A) and a concomitant decrease in anti-inflammatory M2 macrophages in the kidneys of SSHTN mice compared with control kidneys (Figure 2B). As expected, AVE treatment significantly decreased renal M1 macrophages (Figure 2A) while increasing renal M2 macrophages in SSHTN+AVE mice (Figure 2B), demonstrating AVE’s anti-inflammatory effect. Except for male blood levels of M2 macrophages not changing, AVE also significantly increased M2 macrophages in the spleen of male mice and the blood and spleen of female mice (Supplementary Figure S1). Renal dendritic cells (DCs) were significantly increased in SSHTN and SSHTN+AVE mice compared with control mice (Figure 2C). There was a significant increase in natural killer (NK) cells in the kidneys of SSHTN mice compared with those of control mice, which was effectively prevented by AVE treatment (Figure 2D). Moreover, there was an increase in CD4+IFNg+ and CD4+TNFa+ T helper 1 (Th1) cell populations observed in the kidneys of SSHTN mice compared with kidneys from control mice (Figure 2E). AVE treatment mitigated this increase in the kidneys of SSHTN+AVE mice (Figure 2E). SSHTN mice exhibited a significant increase in renal CD4+IL17+ T helper 17 (Th17) cells compared with control mice, while SSHTN+AVE mice had a decrease in renal Th17 cells compared with SSHTN mice (Figure 2E). CD4+CD25+FoxP3+ regulatory T cells (Tregs) and CD4+IL4+ T helper 2 (Th2) cells decreased significantly in the kidneys of SSHTN mice, while these populations were increased in SSHTN+AVE kidneys compared with SSHTN kidneys (Figure 2E).

To expand upon these findings, we utilized real-time quantitative PCR (qRT-PCR) to analyze gene expression of various cytokines. There were significant increases in renal expression of the pro-inflammatory cytokines *Il6* and *Tnfa* in SSHTN mice compared with control mice (Figure 2F). Significant decreases in renal *Il6* and *Tnfa* expression were observed in SSHTN+AVE mice (Figure 2F). There were no significant changes in renal expression levels of *Il1b, Il17, Ifng, Nos2*, or *Il10* (Figure 2F). These findings collectively indicate the effectiveness of AVE in alleviating inflammation associated with SSHTN.

### AVE treatment attenuated renal lymphangiogenesis in male SSHTN mice

**Figure 3:**
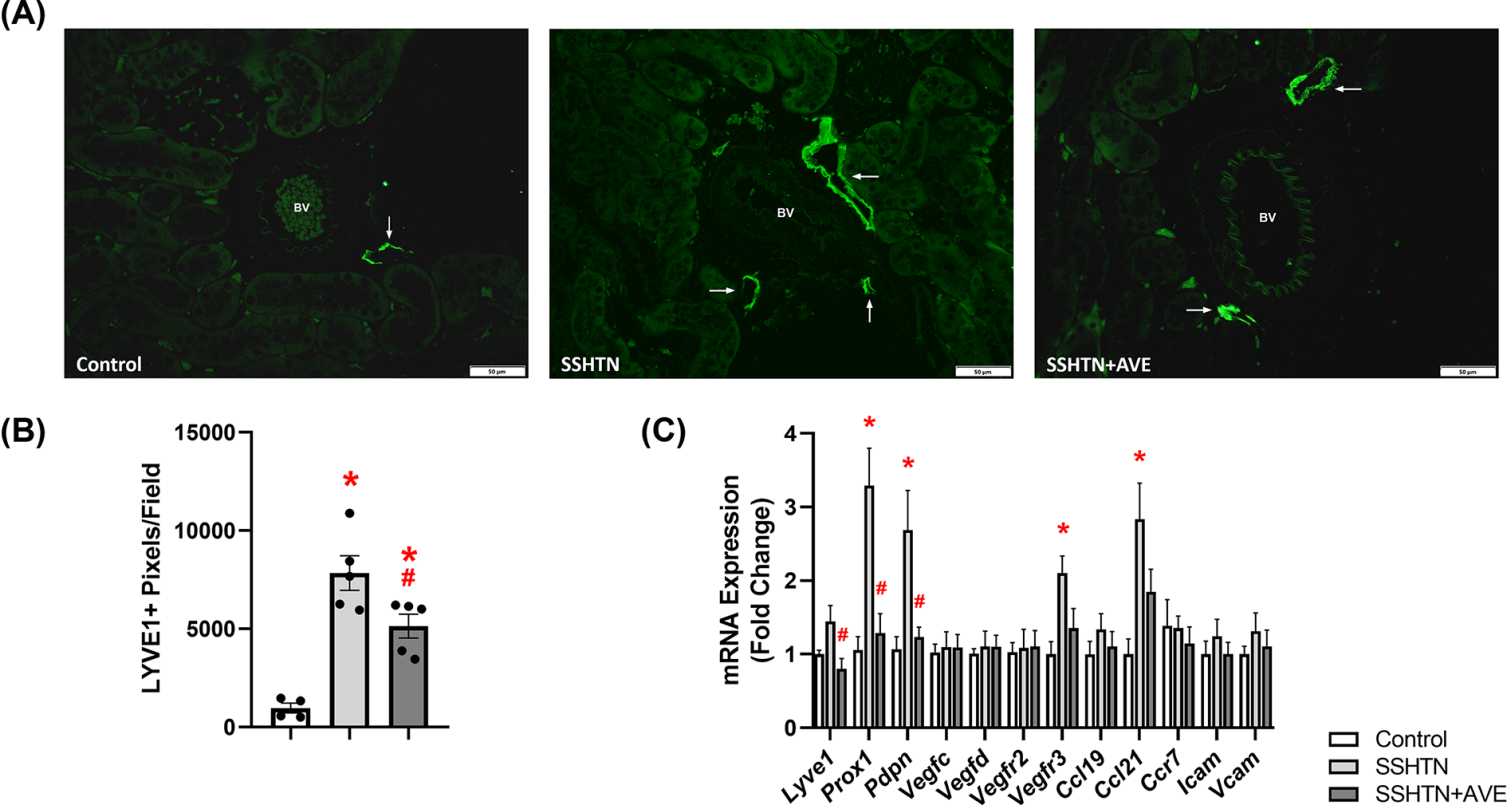
AVE0991 treatment decreased lymphatics in the kidneys of male mice with SSHTN (**A**) LYVE1 immunofluorescence in the kidneys of control, SSHTN, and SSHTN+AVE male mice. LYVE1 is labeled green and lymphatic vessels are indicated with arrows surrounding the blood vessels (BV). Images were taken in the cortex at 20×. Scale bars = 50 µm. (**B**) Renal lymphatic density in control, SSHTN, and SSHTN+AVE male mice as determined by quantification of LYVE1+ pixels per field. (**C**) Renal expression changes in lymphangiogenesis-related genes in control, SSHTN, and SSHTN+AVE male mice. Results are presented as mean ± SEM and all statistical analyses were performed with one-way ANOVA (*n* = 3-5 per group). *P<0.05 vs control mice and #*P*<0.05 vs SSHTN mice.

Considering that SSHTN induces inflammation-associated lymphangiogenesis in the kidneys, we explored the impact of promoting M2 macrophage polarization on renal lymphatics. Kidney sections were immunolabeled with lymphatic endothelial cell marker LYVE1 and imaged. Quantification of LYVE1+ pixels per field revealed a significant increase in renal lymphatic vessel density in SSHTN mice compared with control mice (Figure 3A,B). AVE treatment significantly attenuated renal lymphatic density in SSHTN+AVE mice compared with SSHTN mice (Figure 3A,B). Additionally, gene expression analysis of lymphatic vessel markers showed significant increases in *Prox1, Pdpn, Vegfr3*, and *Ccl21* expression in the kidneys of SSHTN mice compared with kidneys from control mice (Figure 3C). SSHTN+AVE mice had significantly decreased renal expression of *Lyve1, Prox1*, and *Pdpn* compared with SSHTN mice (Figure 3C). AVE treatment prevented the increases in renal *Vegfr3* and *Ccl21* expression levels (Figure 3C). No significant changes were observed in the renal expression of *Vegfc, Vegfd, Vegfr2, Ccl19, Ccr7, Icam*, or *Vcam* (Figure 3C).

### AVE treatment improved renal function in male SSHTN mice

**Figure 4:**
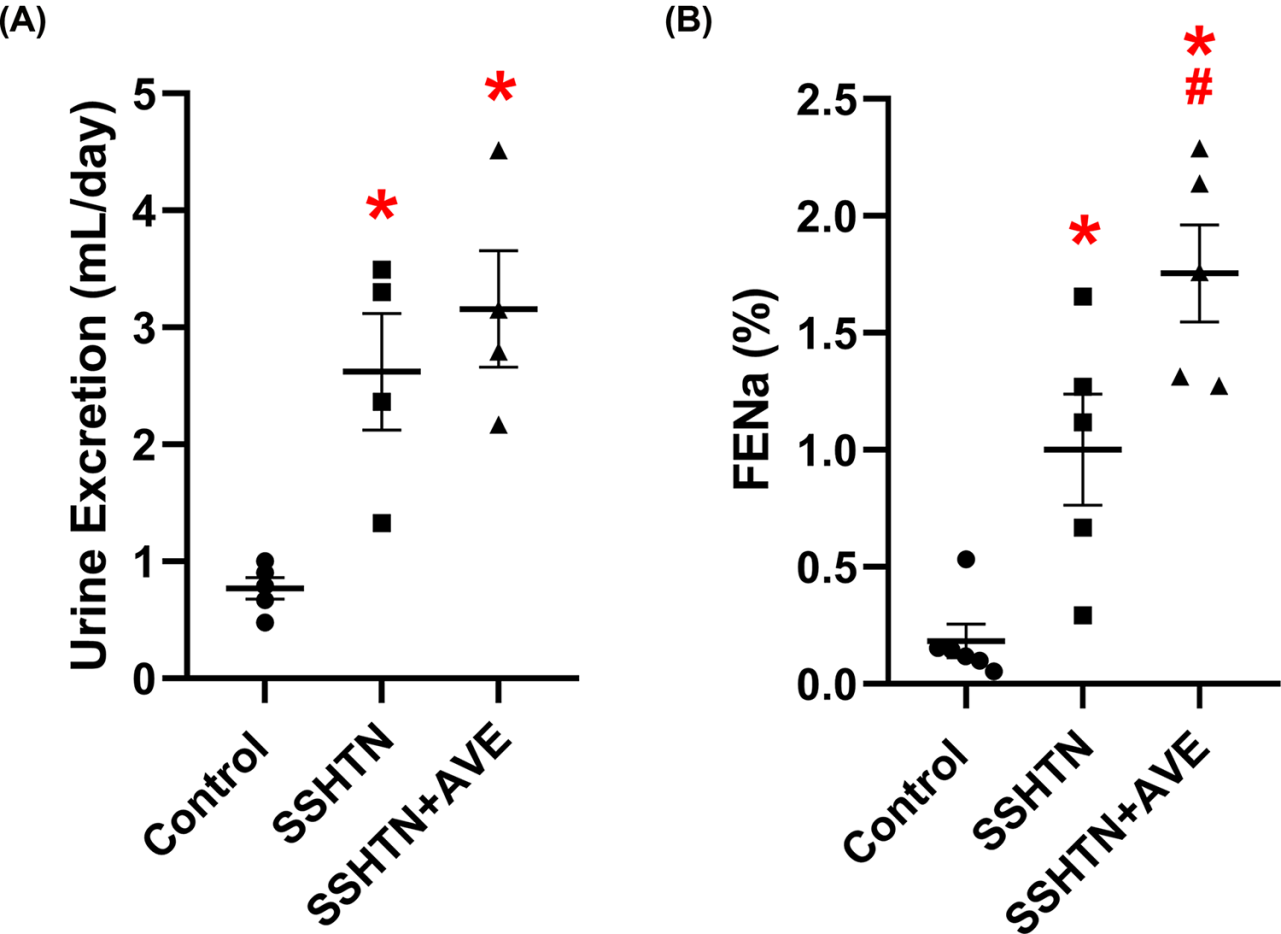
AVE0991 treatment improved kidney function in male mice with SSHTN (**A**) 24-h urine excretion and (**B**) fractional excretion of sodium (FENa) in control, SSHTN, and SSHTN+AVE male mice. Results are presented as mean ± SEM and all statistical analyses were performed with one-way ANOVA (*n* = 4–6 per group). *P<0.05 vs control mice and #*P*<0.05 vs SSHTN mice.

To assess the impact of increasing anti-inflammatory macrophages on renal function, we measured urine output and fractional excretion of sodium (FENa). Both the SSHTN and SSHTN+AVE groups exhibited significant increases in urine output compared with the control group (Figure 4A). Additionally, significant increases in FENa were observed in both the SSHTN and SSHTN+AVE groups compared with the control group (Figure 4B). SSHTN+AVE mice showed a further increase in FENa when compared with SSHTN mice Figure 4B).

### AVE treatment improved testicular immune cells and reduced testicular inflammation in male SSHTN mice

**Figure 5:**
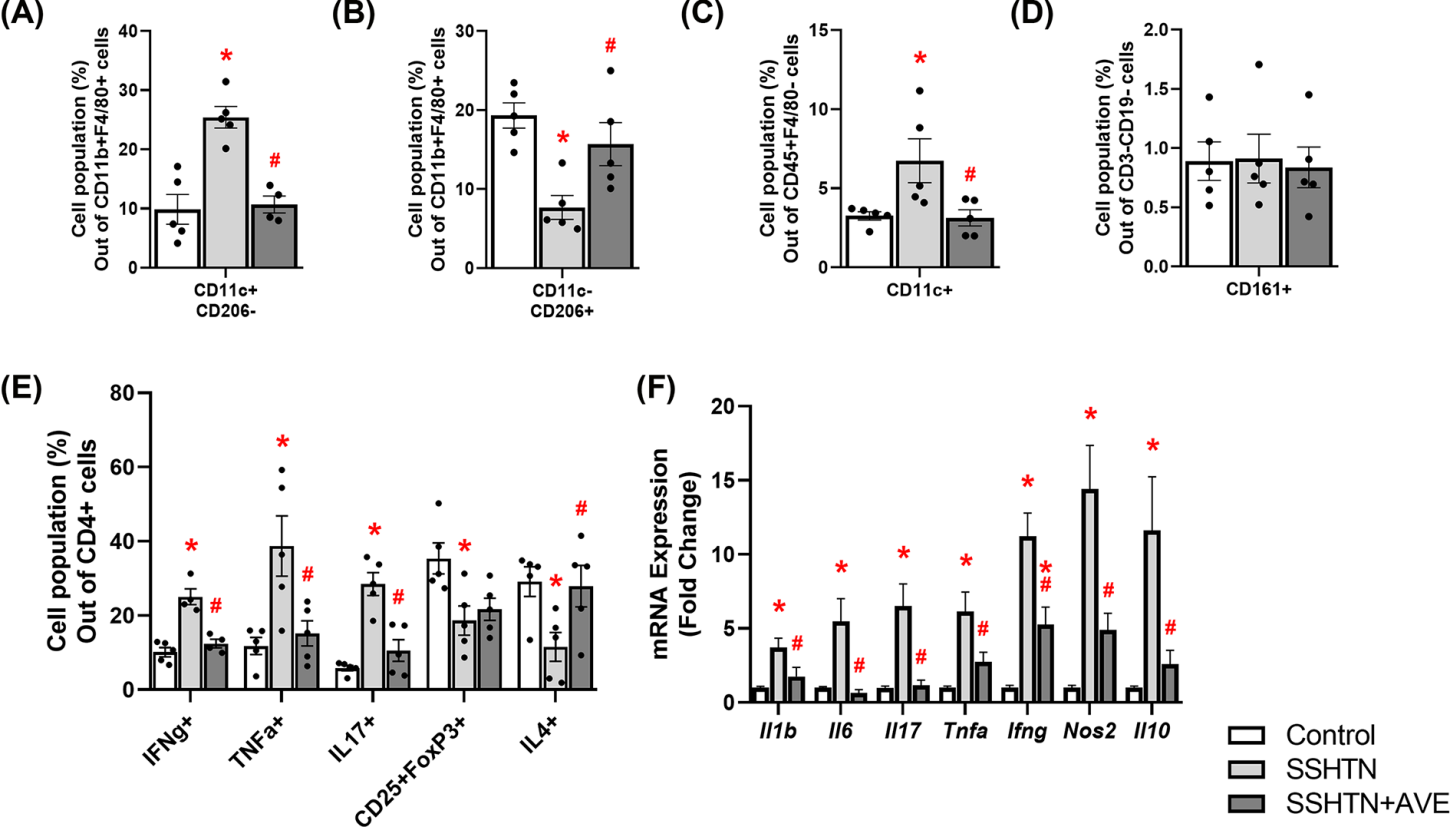
AVE0991 treatment favorably altered immune cells and decreased inflammation in the testes of male mice with SSHTN Flow cytometric data examining testicular populations of (**A**) M1 macrophages, (**B**) M2 macrophages, (**C**) DCs, (**D**) NK cells, and (**E**) T cells in control, SSHTN, and SSHTN+AVE male mice. Each cell population is shown as a percentage of its respective parent population. (**F**) Testicular cytokine expression changes in control, SSHTN, and SSHTN+AVE male mice. Results are presented as mean ± SEM and statistical analyses were performed with one-way ANOVA (*n* = 4-6 per group). *P<0.05 vs control mice and #*P*<0.05 vs SSHTN mice.

Our prior investigation revealed an increase in M1 macrophages and a decrease in M2 macrophages in the testes of SSHTN mice, prompting inquiries into the impact of M2 macrophage polarization on testicular inflammation and tissue damage [31]. We characterized testicular immune cells by flow cytometry and found there was a significant increase in M1 macrophages in the testes of SSHTN mice, and this increase was attenuated in SSHTN+AVE mice (Figure 5A). Conversely, there was a significant decrease in M2 macrophages in SSHTN mice relative to controls, while SSHTN+AVE mice had significantly increased testicular M2 macrophages relative to SSHTN mice (Figure 5B). DCs were significantly increased in the testes of SSHTN mice compared with testes from control mice and significantly decreased in the testes of SSHTN+AVE mice compared with testes from SSHTN mice (Figure 5C). No significant changes were detected in testicular NK cells in either SSHTN or SSHTN+AVE mice (Figure 5D). In the testes of SSHTN mice, there were significant increases in CD4+IFNg+ and CD4+TNFa+ Th1 cells and Th17 cells relative to testes from control mice (Figure 5E). Notably, AVE treatment significantly decreased these pro-inflammatory cell populations compared with SSHTN mice (Figure 5E). Testicular Tregs and Th2 cells were decreased in SSHTN mice compared with controls (Figure 5E). AVE treatment significantly increased testicular Th2 cells relative to SSHTN mice and prevented a significant decrease in Tregs relative to control mice (Figure 5E).

Further exploration of testicular inflammation using qRT-PCR unveiled significant increases in testicular expression of the pro-inflammatory cytokines *Il1b, Il6, Il17, Tnfa, Ifng, Nos2*, and *Il10* in SSHTN mice (Figure 5F). Testicular expression levels of these genes were decreased in SSHTN+AVE mice compared with SSHTN mice (Figure 5F). mRNA expression of *Ifng* was significantly increased in the testes of both the SSHTN and SSHTN+AVE groups compared with control testes (Figure 5F).

### AVE treatment alleviated testicular lymphangiogenesis in male SSHTN mice

**Figure 6:**
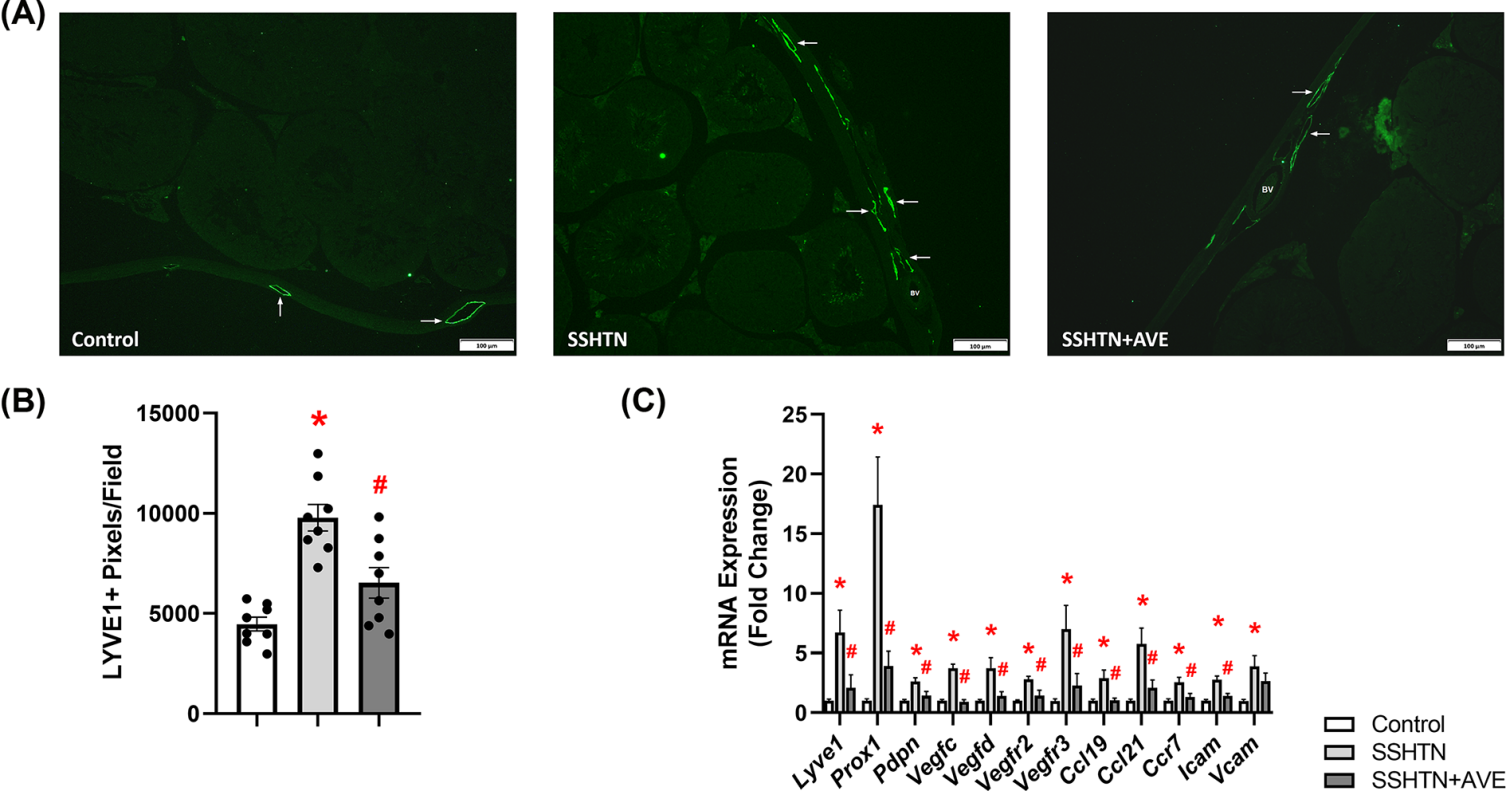
AVE0991 treatment decreased lymphatics in the testes of male mice with SSHTN (**A**) LYVE1 immunofluorescence in the testes of control, SSHTN, and SSHTN+AVE male mice. LYVE1 is labeled green and lymphatic vessels are indicated with arrows. Images were taken in the outer tunica albuginea at 10×. Scale bars = 100 µm. (**B**) Testicular lymphatic density in control, SSHTN, and SSHTN+AVE male mice as determined by quantification of LYVE1+ pixels per field (*n* = 8 per group). (**C**) Testicular expression changes in lymphangiogenesis-related genes in control, SSHTN, and SSHTN+AVE male mice (*n* = 5–7 per group). Results are presented as mean ± SEM and all statistical analyses were performed with one-way ANOVA. *P<0.05 vs control mice and #*P*<0.05 vs SSHTN mice.

Next, we explored how inducing macrophages toward an M2 phenotype impacts testicular lymphatics. Quantification of LYVE1+ pixels revealed a significant increase in lymphatic density within the testes of SSHTN mice when compared with control testes (Figure 6A,B). Compared with SSHTN mice, SSHTN+AVE mice had a significant decrease in testicular lymphatic density (Figure 6A,B). Gene expression analysis further corroborated these findings. Compared with control testes, SSHTN testes demonstrated increased levels of *Lyve1, Prox1*, and *Pdpn*, as well as increased expression of lymphangiogenic growth factors *Vegfc* and *Vegfd* and their receptors *Vegfr2* and *Vegfr3* (Figure 6C). AVE treatment decreased testicular expression of these genes compared with SSHTN mice (Figure 6C). Furthermore, expression levels of the lymphatic-specific chemokines *Ccl19* and *Ccl21*, along with their receptor *Ccr7*, were increased in the testes of SSHTN mice relative to controls (Figure 6C). These genes were decreased significantly in the testes of SSHTN+AVE mice relative to SSHTN testes (Figure 6C). Expression levels of the adhesion molecules *Icam* and *Vcam* were significantly increased in the testes of SSHTN mice compared with control testes, while AVE treatment led to a decrease in *Icam* relative to SSHTN testes and prevented an increase in *Vcam* (Figure 6C).

### AVE treatment partially restored testicular function in male SSHTN mice

**Figure 7:**
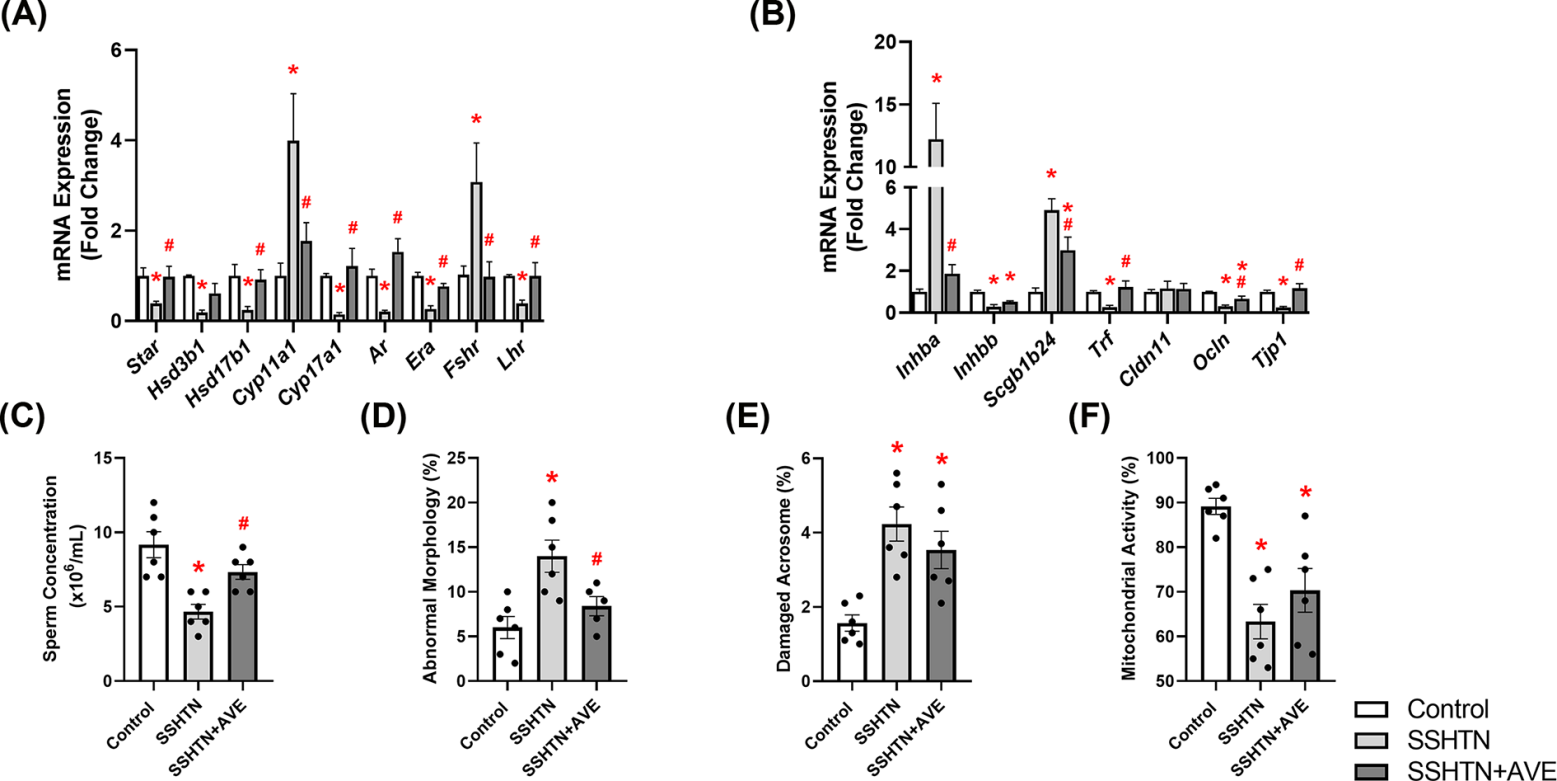
AVE0991 treatment improved testicular function and partially improved sperm health in male mice with SSHTN Testicular expression changes in (**A**) steroidogenic pathway-related genes and hormone receptors and (**B**) secretory and tight junction proteins in control, SSHTN, and SSHTN+AVE male mice (*n* = 4–7 per group). Measures of sperm (**C**) concentrations, (**D**) morphology, (**E**) mitochondrial activity levels, and (**F**) acrosome integrity to assess sperm quality and reproductive health in control, SSHTN, and SSHTN+AVE male mice (*n* = 5–6 per group). Results are presented as mean ± SEM and statistical analyses were performed with one-way ANOVA. *P<0.05 vs control mice and #*P*<0.05 vs SSHTN mice.

Our subsequent aim was to investigate whether promoting M2 macrophage polarization could reverse testicular damage in SSHTN. Therefore, we analyzed mRNA expression patterns of steroidogenic pathway genes, hormone receptors, secretory proteins, and tight junction proteins by qRT-PCR. We observed significant decreases in the expression of *Star, Hsd3b1*, and *Hsd17b1* in the testes of SSHTN mice compared with control testes (Figure 7A). AVE treatment was associated with significant increases in testicular *Star* and *Hsd17b1* expression levels relative to SSHTN mice, and prevented a decrease in *Hsd3b1* (Figure 7A). *Cyp11a*1 expression was increased in the testes of SSHTN mice compared wiith testes of control mice, whereas SSHTN+AVE mice exhibited a decrease in testicular *Cyp11a*1 expression compared with SSHTN mice (Figure 7A). A significant decrease in *Cyp17a1* expression was observed in the testes of SSHTN mice compared with control testes, while *Cyp17a1* expression was increased in the testes of SSHTN+AVE mice compared with SSHTN testes (Figure 7A). Additionally, testes from SSHTN mice exhibited decreases in the hormone receptors *Ar, Era*, and *Lhr*, which were successfully mitigated by AVE treatment (Figure 7A). Expression of *Fshr* was increased in the testes of SSHTN mice relative to control testes and decreased in the testes of SSHTN+AVE mice relative to SSHTN testes (Figure 7A). Moreover, we observed significant increases in *Inhba* and *Scgb1b24* in the testes of SSHTN mice compared with control testes (Figure 7B). Testicular expression of these genes was decreased in SSHTN+AVE testes compared with SSHTN testes, although *Scgb1b24* remained increased in SSHTN+AVE testes compared with control testes (Figure 7B). There was a significant decrease in the expression of *Inhbb* in the testes of SSHTN and SSHTN+AVE mice compared with testes of control mice (Figure 7B). SSHTN mice exhibited significant decreases in testicular expression of *Trf, Ocln*, and *Tjp1* compared with control mice (Figure 7B). Testicular expression levels of *Trf* and *Tjp1* were significantly increased in SSHTN+AVE mice compared with SSHTN mice, bringing them closer to control levels (Figure 7B). Testicular *Ocln* expression was significantly increased in SSHTN+AVE mice relative to SSHTN mice but remained significantly decreased compared with control mice (Figure 7B). Testicular *Cldn11* expression remained unchanged in both experimental groups (Figure 7B).

To explore potential improvements in testicular function in AVE-treated SSHTN mice, we evaluated various sperm parameters including concentration, morphology, acrosome integrity, and mitochondrial activity. SSHTN mice demonstrated decreased sperm concentration, which was rescued by AVE treatment (Figure 7C). SSHTN mice displayed a significant increase in the percentage of sperm exhibiting abnormal morphology, whereas SSHTN+AVE mice showed a decrease in abnormal sperm compared with SSHTN mice (Figure 7D). Both SSHTN and SSHTN+AVE groups exhibited increases in sperm with damaged acrosomes relative to the control group (Figure 7F). SSHTN and SSHTN+AVE mice also demonstrated decreases in the percentage of sperm with functional mitochondria compared with control mice (Figure 7F).

### AVE treatment attenuated SBP, renal pro-inflammatory immune cells, and renal inflammation in female SSHTN mice

**Figure 8:**
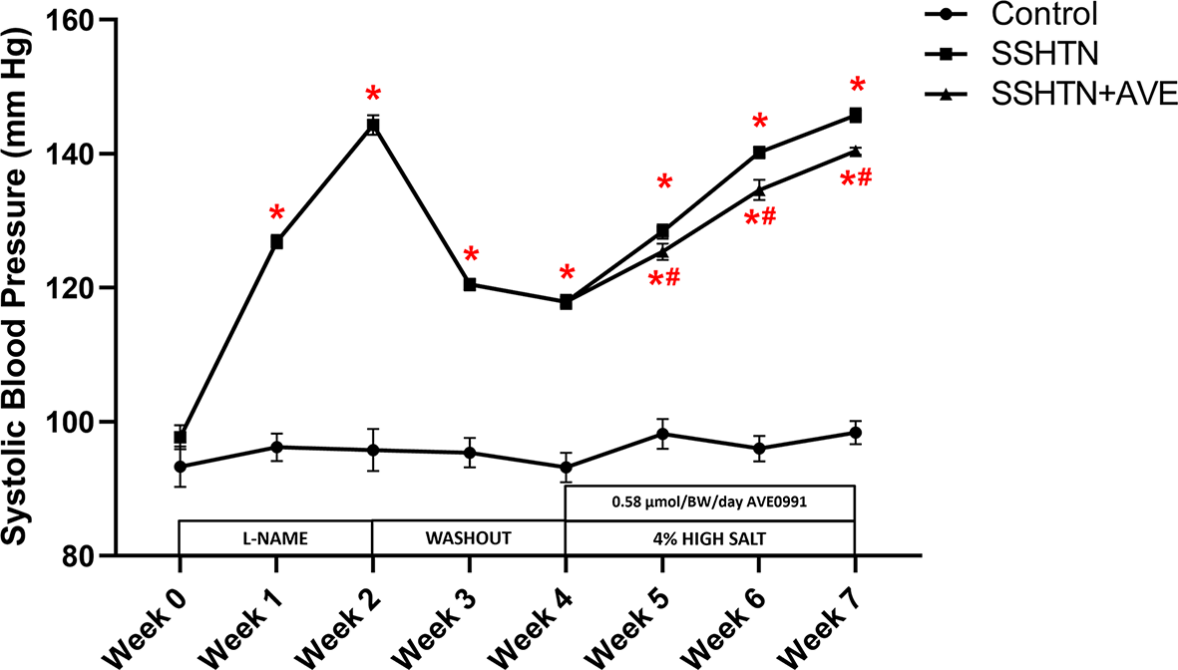
AVE0991 treatment attenuated SBP in female mice with SSHTN SBP measurements in untreated control, SSHTN, and SSHTN+AVE female mice. Blood pressures were taken weekly via tail cuff. Results are presented as mean ± SEM and statistical analyses were performed with one-way ANOVA (*n* = 5 per group). *P<0.05 vs control mice and #*P*<0.05 vs SSHTN mice.

Our subsequent objective was to determine whether administering AVE could influence macrophage polarization in the kidneys and lower BP in female SSHTN mice. Following previous findings, SSHTN mice had a significant increase in SBP compared with control mice for each week (Figure 8). This effect was attenuated by the administration of AVE as SSHTN+AVE mice had decreased SBP compared with SSHTN mice each week of the high salt diet phase, although these values remained significantly increased compared with those of control mice (Figure 8).

**Figure 9:**
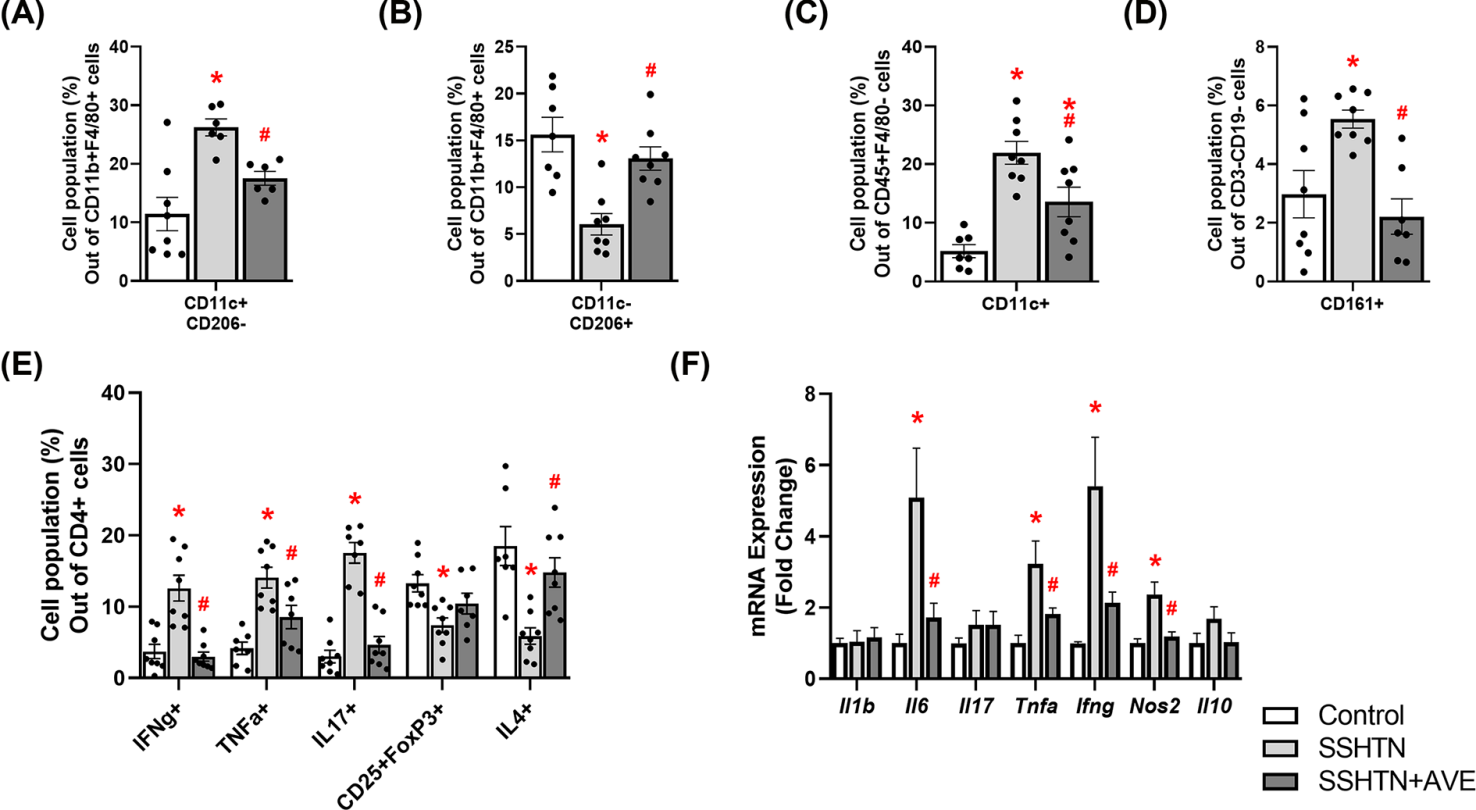
AVE0991 treatment attenuated immune cells and inflammation in the kidneys of female mice with SSHTN Flow cytometric data examining renal populations of (**A**) M1 macrophages, (**B**) M2 macrophages, (**C**) DCs, (**D**) NK cells, and (**E**) T cells in control, SSHTN, and SSHTN+AVE female mice (*n* = 4–5 per group). Each cell population is shown as a percentage of its respective parent population. (**F**) Renal cytokine expression changes in control, SSHTN, and SSHTN+AVE female mice (*n* = 3–6 per group). Results are presented as mean ± SEM and statistical analyses were performed with one-way ANOVA. *P<0.05 vs control mice and #*P*<0.05 vs SSHTN mice.

Flow cytometric analysis revealed an increase in M1 macrophages (Figure 9A) and a corresponding decrease in M2 macrophages within the kidneys of SSHTN mice compared with control kidneys (Figure 9B). SSHTN+AVE kidneys exhibited a significant decrease in M1 macrophages (Figure 9A) and an increase in M2 macrophages compared with SSHTN kidneys (Figure 9B). Renal DCs were increased in both of the SSHTN groups compared with controls but were decreased significantly in the SSHTN+AVE group compared with the SSHTN group (Figure 9C). The kidneys of SSHTN mice exhibited an increase in NK cells compared with control kidneys, and this change was mitigated by AVE treatment (Figure 9D). CD4+IFNg+ and CD4+TNFa+ Th1 cells, as well as Th17 cells, were increased significantly in the kidneys of SSHTN mice compared with control kidneys (Figure 9E). These populations were decreased in SSHTN+AVE mice compared with SSHTN mice (Figure 9E). Renal Tregs and Th2 cells were decreased significantly in SSHTN mice compared with control mice, whereas AVE treatment significantly increased renal Th2 cells compared with SSHTN mice (Figure 9E).

Analysis of cytokine gene expression further corroborated these findings, revealing increased mRNA expression levels of *Il6, Tnfa, Ifng*, and *Nos2* in the kidneys of SSHTN mice compared with the kidneys of control mice (Figure 9F). These genes were decreased significantly by AVE treatment compared with SSHTN mice (Figure 9F). There were no significant alterations observed in renal expression of *Il1b, Il17*, or *Il10* (Figure 9F).

### AVE treatment differentially affected renal lymphangiogenesis in female SSHTN mice

**Figure 10:**
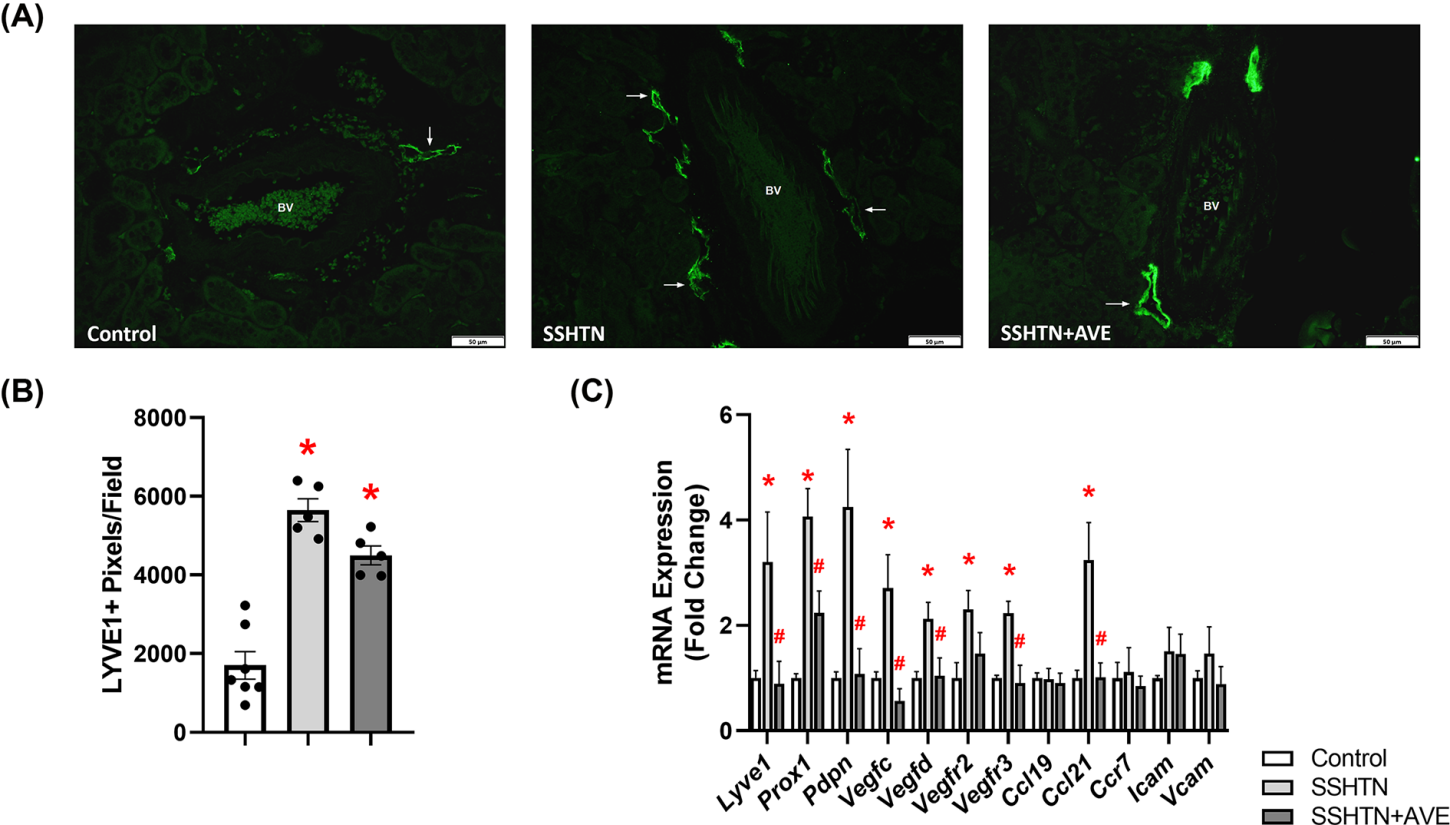
AVE0991 treatment differentially affected lymphatics in the kidneys of female mice with SSHTN (**A**) LYVE1 immunofluorescence in the kidneys of control, SSHTN, and SSHTN+AVE female mice. LYVE1 is labeled green and lymphatic vessels are indicated with arrows surrounding the blood vessels (BV). Images were taken in the cortex at 20×. Scale bars = 50 µm. (**B**) Renal lymphatic density in control, SSHTN, and SSHTN+AVE female mice as determined by quantification of LYVE1+ pixels per field (*n* = 5–7 per group). (**C**) Renal expression changes in lymphangiogenesis-related genes in control, SSHTN, and SSHTN+AVE female mice (*n* = 4–6 per group). Results are presented as mean ± SEM and all statistical analyses were performed with one-way ANOVA. *P<0.05 vs control mice and #*P*<0.05 vs SSHTN mice.

In our investigation of female mice, we delved into potential sex-specific differences in renal lymphatic density in response to macrophage modulation by AVE treatment. Analysis of LYVE1+ staining revealed significant increases in renal lymphatic density in both the SSHTN and SSHTN+AVE groups compared with the control group (Figure 10A,B). These findings were in contrast with gene expression analyses. Significant increases in *Lyve1, Prox1, Pdpn, Vegfc, Vegfd, Vegfr2, Vegfr3*, and *Ccl21* were observed in the kidneys of SSHTN mice compared with control kidneys, and expression levels of these genes were decreased in SSHTN+AVE kidneys compared with SSHTN kidneys, with the exception of *Vegfr2* (Figure 10C). There were no alterations observed in the expression levels of *Ccl19, Ccr7, Icam*, or *Vcam* (Figure 10C).

### AVE treatment did not improve renal function in female SSHTN mice

**Figure 11:**
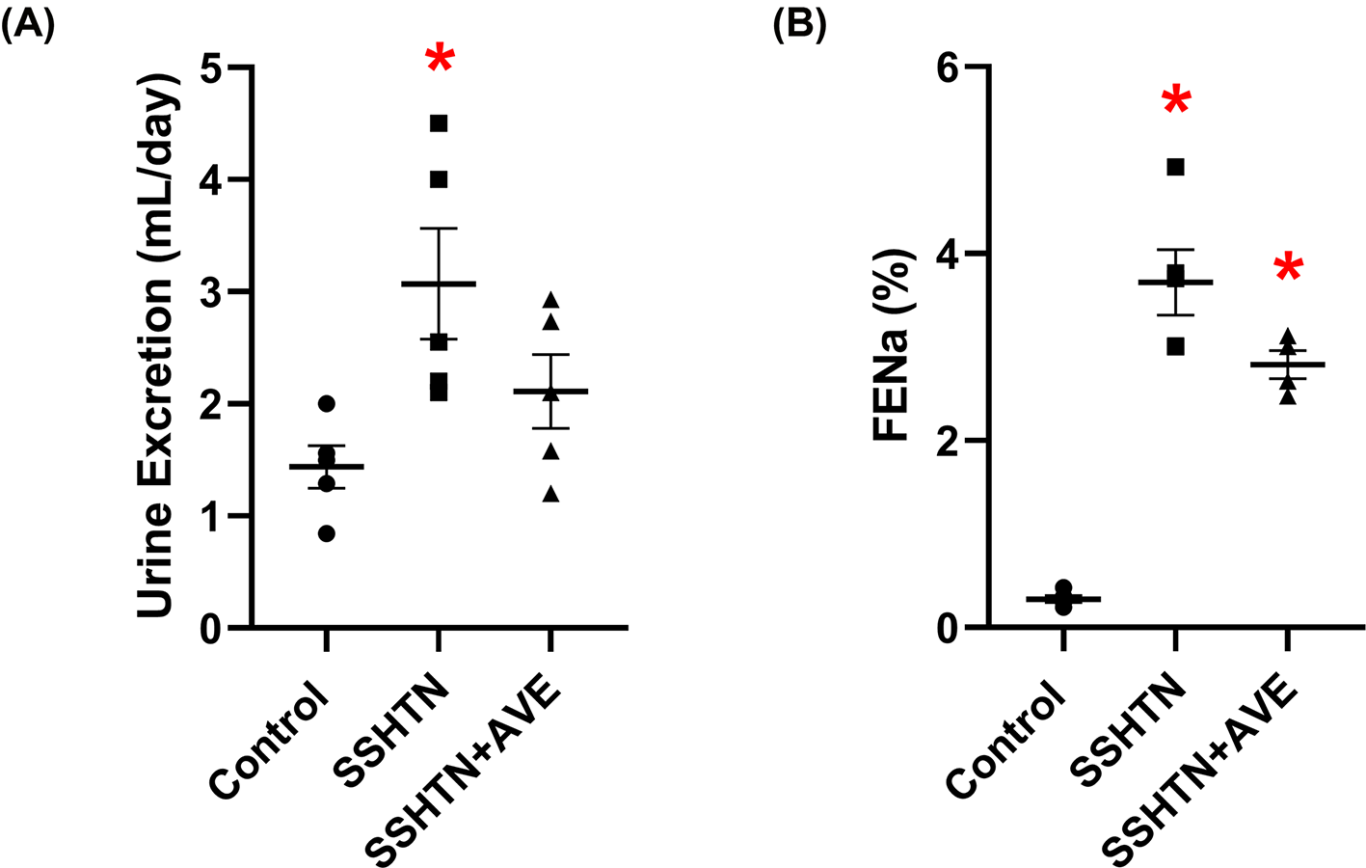
AVE0991 treatment did not improve kidney function in female mice with SSHTN (**A**) 24-h urine excretion and (**B**) fractional excretion of sodium (FENa) in control, SSHTN, and SSHTN+AVE female mice. Results are presented as mean ± SEM and all statistical analyses were performed with one-way ANOVA s (*n* = 4–5 per group). *P<0.05 vs control mice and #*P*<0.05 vs SSHTN mice.

We then evaluated renal function in female SSHTN mice after AVE-induced M2 macrophage polarization. We observed a significant increase in urine output in SSHTN mice compared with control mice (Figure 11A). SSHTN+AVE mice did not experience any change in urine output (Figure 11A). Both SSHTN and SSHTN+AVE groups demonstrated significant increases in FENa relative to the control group (Figure 11B).

### AVE treatment differentially impacted ovarian immune cells and inflammation in female SSHTN mice

**Figure 12:**
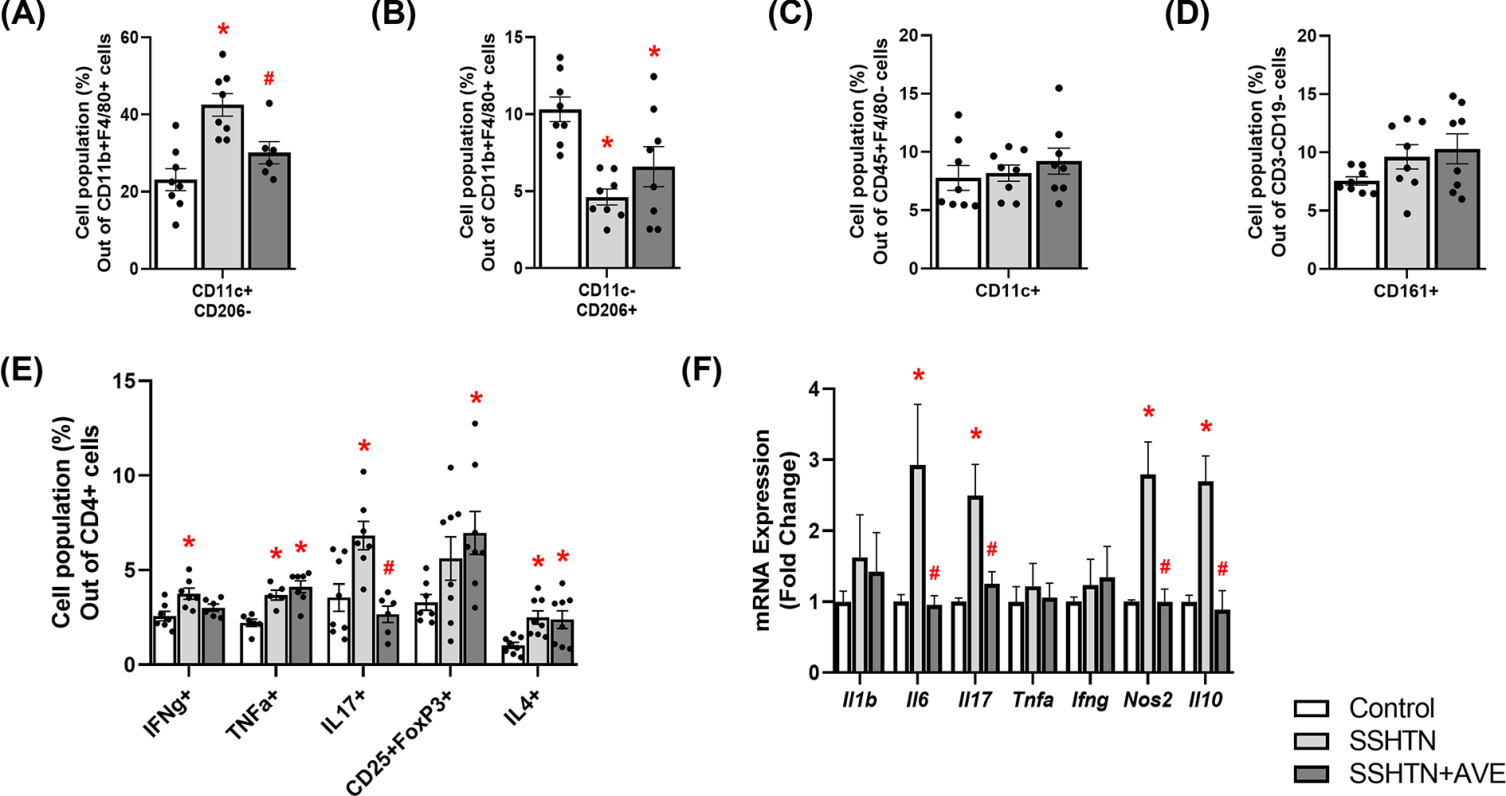
AVE0991 treatment differentially affected immune cells and inflammation in the ovaries of female mice with SSHTN Flow cytometric data examining ovarian populations of (**A**) M1 macrophages, (**B**) M2 macrophages, (**C**) DCs, (**D**) NK cells, and (**E**) T cells in control, SSHTN, and SSHTN+AVE female mice (*n* = 4–5 per group). Each cell population is shown as a percentage of its respective parent population. (**F**) Ovarian cytokine expression changes in control, SSHTN, and SSHTN+AVE female mice (*n* = 5–6 per group). Results are presented as mean ± SEM and statistical analyses were performed with one-way ANOVA. *P<0.05 vs control mice and #*P*<0.05 vs SSHTN mice.

Analysis of ovarian cell suspensions using flow cytometry revealed a significant increase in M1 macrophages in SSHTN ovaries relative to control ovaries and a decrease in M1 macrophages in SSHTN+AVE ovaries relative to SSHTN ovaries (Figure 12A). A concurrent decrease in ovarian M2 macrophages was observed in both SSHTN and SSHTN+AVE mice when compared with control mice (Figure 12B). Ovarian DCs and NK cells were unaltered in both the SSHTN and SSHTN+AVE groups (Figure 12C,D). There were increases in ovarian CD4+IFNg+ Th1 cells and Th17 cells in SSHTN mice relative to control mice (Figure 12E). In the SSHTN+AVE group, ovarian Th17 cells decreased significantly and ovarian CD4+IFNg+ Th1 cells trended toward a decrease compared with the SSHTN group (Figure 12E). Th2 cells and CD4+TNFa+ Th1 cells were increased significantly in the ovaries of both SSHTN and SSHTN+AVE mice compared with control ovaries (Figure 12E). SSHTN+AVE ovaries exhibited an increase in Tregs compared with control ovaries (Figure 12E).

Subsequently, we investigated gene expression profiles to gain deeper insights into the effect of AVE treatment on ovarian inflammation. qRT-PCR analysis revealed significant increases in *Il6, Il17, Nos2*, and *Il10* expression in the ovaries of SSHTN mice relative to control ovaries (Figure 12F). Ovarian expression levels of these genes were decreased significantly in SSHTN+AVE mice compared with SSHTN mice (Figure 12F). There were no changes in ovarian expression levels of *Il1b, Tnfa*, or *Ifng* (Figure 12F).

### AVE treatment alleviated ovarian lymphatic density in female SSHTN mice

**Figure 13:**
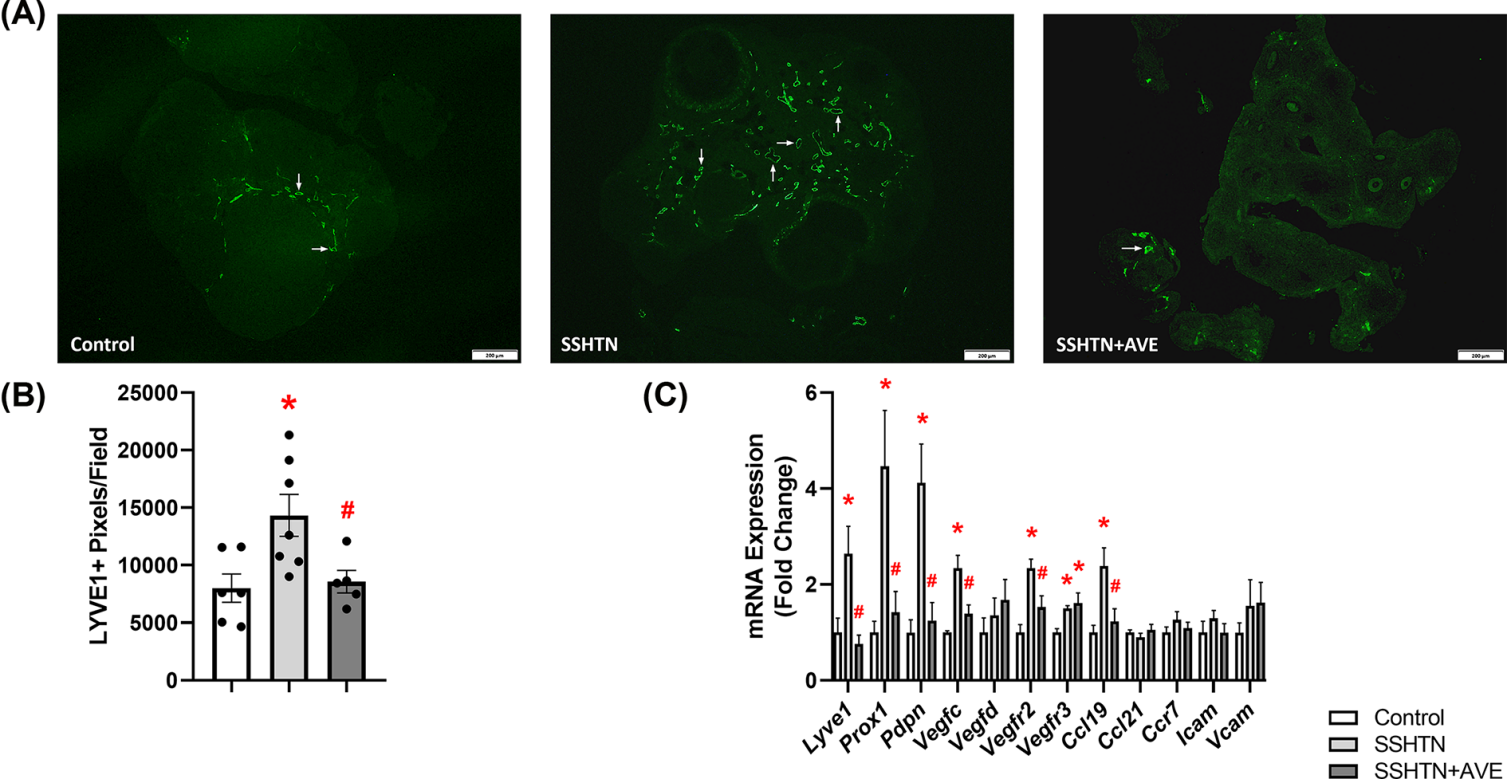
AVE0991 treatment decreased lymphangiogenesis in the ovaries of female mice with SSHTN (**A**) LYVE1 immunofluorescence in the ovaries of control, SSHTN, and SSHTN+AVE female mice. LYVE1 is labeled green and lymphatic vessels are indicated with arrows. Images were taken of the whole ovary at 4×. Scale bars = 200 µm. (**B**) Ovarian lymphatic density in control, SSHTN, and SSHTN+AVE female mice as determined by quantification of LYVE1+ pixels per field (*n* = 5–7 per group). (**C**) Ovarian expression changes in lymphangiogenesis-related genes in control, SSHTN, and SSHTN+AVE female mice (*n* = 4–5 per group). Results are presented as mean ± SEM and all statistical analyses were performed with one-way ANOVA. *P<0.05 vs control mice and #*P*<0.05 vs SSHTN mice.

To determine whether alterations in macrophage polarization can impact ovarian lymphatics, we immunostained ovarian sections with LYVE1. Quantification of LYVE1+ pixels revealed ovarian lymphatic density was increased significantly in SSHTN mice relative to control mice and decreased significantly in SSHTN+AVE mice relative to SSHTN mice (Figure 13A,B). Additionally, there were significant increases in ovarian expression of *Lyve1, Prox1, Pdpn, Vegfc, Vegfr2*, and *Ccl19* in SSHTN mice compared with controls, and these changes were attenuated by AVE treatment (Figure 13C). Ovaries in both the SSHTN and SSHTN+AVE groups had increased *Vegfr3* expression (Figure 13C). No changes were observed in the ovarian expression levels of *Vegfd, Ccl21, Ccr7, Icam*, or *Vcam*. (Figure 13C).

### AVE treatment improved ovarian function in female SSHTN mice

**Figure 14:**
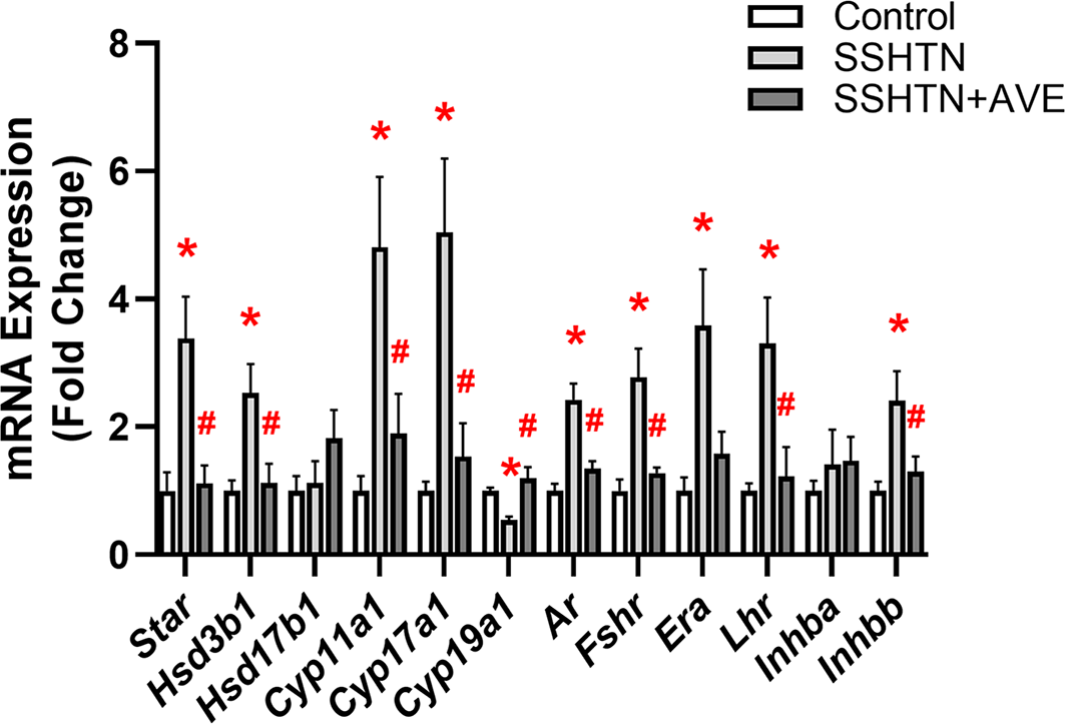
AVE0991 treatment improved ovarian function in female mice with SSHTN Ovarian expression changes in cholestrol transport proteins, steroidogenic enzymes, and hormone receptors in control, SSHTN, and SSHTN+AVE female mice. Results are presented as mean ± SEM and statistical analyses were performed with one-way ANOVA (*n* = 4-5 per group). *P<0.05 vs control mice and #*P*<0.05 vs SSHTN mice.

Next, we examined the impact of inducing macrophages toward an M2 phenotype on ovarian function. Gene expression analysis unveiled significant increases in the expression of *Star, Hsd3b1, Cyp11a1*, and *Cyp17a1* in SSHTN ovaries compared with control ovaries. Treatment with AVE significantly decreased ovarian expression of these genes compared with SSHTN mice (Figure 14). Expression of aromatase enzyme *Cyp19a1* was decreased significantly in the ovaries of SSHTN mice compared with control ovaries and increased significantly in SSHTN+AVE ovaries compared with SSHTN ovaries (Figure 14). In the ovaries of SSHTN mice, there were significant increases in *Ar, Fshr, Era*, and *Lhr* compared with control ovaries (Figure 14). Ovarian expression levels of *Ar, Fshr*, and *Lhr* were attenuated by AVE treatment, while ovarian *Era* expression trended toward a decrease in SSHTN+AVE mice compared with SSHTN mice (Figure 14). *Inhbb* expression was increased significantly in the ovaries of SSHTN mice compared with control ovaries and decreased significantly in the ovaries of SSHTN+AVE mice compared with SSHTN ovaries (Figure 14). No significant changes were observed in ovarian *Hsd17b1* or *Inhba* expression levels (Figure 14).

## Discussion

Macrophages play a pivotal role in regulating inflammation and maintaining tissue homeostasis. They are polarized into distinct phenotypes, including pro-inflammatory M1 and anti-inflammatory M2 states, and a variety in between, which influence the progression and resolution of inflammation [45]. SSHTN is associated with an increase in M1 macrophages and inflammation resulting in damage to various organs, including kidneys and gonads [29,31]. Nonetheless, the impact of inducing macrophage polarization toward the M2 phenotype on BP, inflammation, and lymphangiogenesis under SSHTN conditions was unknown. The current study investigated whether AVE-induced M2 macrophage polarization could alleviate the BP elevation and the inflammation and end organ damage in kidneys and gonads of mice with SSHTN. By modulating macrophage polarization, the study assessed the therapeutic potential of AVE in mitigating the pathological processes associated with SSHTN. Data from the present study demonstrate that AVE treatment improved BP, inflammation, and immune cell populations and differentially impacted lymphatic density and organ function in the kidneys and gonads of male and female mice with SSHTN.

The observed increase in SBP in SSHTN mice compared with control mice aligns with previous findings [29,31,42–44,46]. The significant reduction in SBP observed in AVE-treated SSHTN mice is consistent with previous studies demonstrating the anti-hypertensive effects of AVE [47–50]. By corroborating and expanding upon prior evidence, our study contributes to a growing body of literature affirming the potential of AVE as a therapeutic agent for reducing SSHTN. However, while our results align with previous studies, it’s important to note that SBP levels in AVE-treated SSHTN mice remained significantly higher than those of control mice. This indicates that while AVE treatment may partially mitigate the HTN phenotype associated with SSHTN, it does not fully normalize BP. Overall, these findings highlight the potential therapeutic benefit of AVE in reducing SBP levels in SSHTN, albeit with some limitations that warrant further investigation and optimization of treatment, like combination therapies, or additional interventions as described in previous studies [50].

Flow cytometric analyses revealed an increase in M1 macrophages and a decrease in M2 macrophages in the kidneys and gonads of SSHTN mice compared with those of control mice, which underscores the inflammatory milieu associated with end organ damage. The decrease of M1 macrophages and augmentation of M2 macrophages observed following AVE treatment demonstrates the drug’s potent anti-inflammatory effect. When polarized *in vitro*, bone marrow-derived macrophages (BMDMs) derived from Mas-deficient mice exhibited increased expression of M1 macrophage markers and reduced expression of M2 macrophage markers compared with BMDMs derived from wild-type mice [51]. Furthermore, pharmacologically activating Mas with AVE attenuated M1 gene expression while enhancing M2 gene expression in BMDMs from Mas-deficient mice, highlighting the capacity of AVE to induce a shift in macrophage polarization towards an anti-inflammatory M2 state [51]. In the present study, AVE exhibited anti-inflammatory properties by influencing the polarization of macrophages toward an M2 phenotype [39]. The observed increase in M2 macrophages and decrease in M1 macrophages following AVE treatment aligns with previous findings, indicating a consistent pattern of AVE-induced modulation of macrophage polarization towards an anti-inflammatory M2 phenotype. Besides affecting macrophages, the administration of AVE resulted in significant decreases in DCs in SSHTN testes and female SSHTN kidneys, NK cells in male and female SSHTN kidneys, and pro-inflammatory T cells in SSHTN testes and female SSHTN kidneys. While Mas receptors are well established on macrophages, their expression on other immune cells like DCs and T cells is less characterized. Notably, Mas receptors have been detected in DCs derived from rat bone marrow [52,53], but their functional significance remains unclear. Given that macrophages can influence T-cell responses and other immune cell functions through cytokine production and antigen presentation, AVE’s effects on macrophage polarization may indirectly impact broader immune responses, as observed in previous studies [51]. These findings suggest that AVE may play a broader regulatory role beyond macrophage polarization, potentially modulating immune responses in SSHTN.

In our study, we observed that AVE generally reduces inflammation in the kidneys and gonads of SSHTN mice by inducing an M2 phenotype in macrophages and suppressing the M1 phenotype, which aligns with previous findings [39]. A previous report demonstrated that augmenting the M2 macrophage population diminishes the M1/M2 ratio, leading to blood pressure normalization in hypertensive rats [25]. Furthermore, the activation of MAS receptors has been associated with enhanced renal function, reduced inflammation, and mitigation of renal damage in a murine model of kidney injury [54]. Associated decreases in pro-inflammatory immune cells, such as DCs, NK cells, and T cells, further contribute to its anti-inflammatory effects. Additionally, AVE treatment has been shown to inhibit oxidative stress, reduce the production of reactive oxygen species, and suppress the expression of inflammatory mediators [39,55]. The BP-lowering effects of MAS receptor activation, as measured by tail-cuff method in the current study, have secondary implications beyond macrophage modulation. Specifically, the reduction in BP might have contributed to a decrease in pro-inflammatory immune cells and inflammation in end organs, an aspect that should not be overlooked. These combined mechanisms highlight the efficacy of AVE in mitigating inflammation in SSHTN, offering promising therapeutic potential for alleviating HTN-induced inflammation and protecting against end organ damage. Further investigation into the precise molecular mechanisms underlying the anti-inflammatory effects of AVE is warranted to optimize its therapeutic application in managing inflammatory conditions associated with SSHTN.

In the current study, there was a significant impact of AVE on cytokine expression in the whole kidney, as compared with individual immune cell subsets that likely stems from the involvement of diverse cell types within renal tissue that are capable of cytokine production and express Mas receptor. Indeed, renal cells such as tubular epithelial cells, mesangial cells, and endothelial cells, possess the ability to produce cytokines in response to stimuli such as inflammation or tissue injury [56,57]. Moreover, Mas receptor expression extends beyond macrophages to include renal tubular and glomerular cells as well as vascular endothelial cells [58–61]. Therefore, the cumulative response of these various cell types to AVE-induced Mas receptor activation might have contributed significantly to overall reduction in cytokine expression within the whole kidney than individual immune cells.

The current findings further underscore the therapeutic potential of AVE in modulating lymphatic vessel density under hypertensive conditions. Specifically, the observed decrease in inflammation-associated lymphangiogenesis in the gonads of both male and female SSHTN mice and the kidneys of male SSHTN mice following AVE treatment highlights the efficacy of this intervention in reducing inflammation-associated lymphangiogenesis. Macrophages, through the release of lymphangiogenic factors like VEGF-C and VEGF-D, have been identified as crucial mediators of lymphatic vessel formation, particularly during inflammation [62–65]. Studies have shown a strong association between the presence of VEGF-C-expressing macrophages and increased lymphatic vessel density [66]. Experimental models of HTN and various inflammatory conditions have consistently revealed inflammation-driven lymphangiogenesis [29,31,42,43,67–69]. In a study utilizing a mouse model of tail lymphedema, it was found that CD68+ macrophages served as source of VEGF-C [70]. Additionally, the removal of macrophages led to a decrease in inflammation-associated lymphangiogenesis in a mouse model of acute colitis [71]. Furthermore, *in vitro* studies have shown that activated murine peritoneal CD11b+ macrophages have the capability to generate tube-like structures and express markers specific to lymphatic endothelial cells [64]. These findings collectively support the notion that activated macrophages and their inflammatory mediators play a significant role in inflammation-associated lymphangiogenesis, which occurs in a compensatory manner to aid in the removal of pro-inflammatory immune cells and resolution of organ inflammation. Therefore, the observed reduction in lymphatic density in AVE-treated SSHTN mice in the current study may be attributed to a decrease in pro-inflammatory macrophages and/or an increase in anti-inflammatory macrophages.

The improvements in renal function observed in male AVE-treated SSHTN mice and gonadal function observed in male and female AVE-treated SSHTN mice can be attributed to the combined effects of reduced BP and inflammation. SSHTN has been shown to be associated with functional impairment and tissue damage in both kidneys and gonads [29,31]. AVE treatment effectively mitigated BP elevation in SSHTN mice, thereby alleviating the hemodynamic burden on the kidneys and gonads, which could have contributed to functional improvement. Moreover, AVE exhibited potent anti-inflammatory properties, as evidenced by the suppression of pro-inflammatory immune cells and cytokines and augmentation of M2 macrophages. Inflammation plays a central role in the pathogenesis of HTN-induced organ damage, including renal and gonadal dysfunction [29,31]. By attenuating inflammation, AVE reduced the release of pro-inflammatory mediators and the infiltration of immune cells into renal and gonadal tissues, thereby preserving tissue integrity and function. Additionally, the reduction in inflammation-associated lymphangiogenesis of AVE-treated SSHTN mice may have resulted from improvements in organ function. Dysfunctional lymphangiogenesis, often observed in inflammatory conditions, can exacerbate tissue damage by inducing immune cell accumulation and fluid accumulation [72]. By suppressing inflammation-associated lymphatic vessel formation, AVE contributed to the maintenance of tissue homeostasis and function. Overall, the observed enhancements in renal function in male AVE-treated SSHTN mice and gonadal function in male and female AVE-treated SSHTN mice underscore the importance of targeting both BP regulation and inflammation to manage SSHTN-induced organ damage.

Mas receptors play a critical role in spermatogenesis and steroidogenesis in the testis [73–75]. Within the ovaries, Mas receptors are pivotal for follicular development, oocyte maturation, and steroidogenesis [76–78]. They also regulate uterine function during pregnancy, including implantation, decidualization, and myometrial contractility [78]. Additionally, Mas receptors influence placental development and function, impacting fetal growth and development [78]. However, research on the effect of AVE on the testes and ovaries is very limited. In diabetic rodent models, AVE significantly enhanced erectile and endothelial function [79]. Furthermore, another study reported the protective effect of AVE on ovarian function in a rat model of polycystic ovary syndrome, showing reductions in ovarian oxidative stress, improvements in follicular development, and restoration of estrous cyclicity [80]. These findings suggest a potential therapeutic application of AVE in ameliorating reproductive dysfunction which is consistent with our results.

Collectively, the results of the present study demonstrate the therapeutic potential of AVE in mitigating inflammation and end organ damage associated with SSHTN. AVE treatment effectively reduced BP, decreased pro-inflammatory immune cell populations, and promoted anti-inflammatory M2 macrophage polarization in the kidneys and gonads of male and female mice with SSHTN. Moreover, AVE attenuated inflammation-associated lymphangiogenesis and improved function in SSHTN gonads and contributed to improvements in lymphatic density in SSHTN kidneys, highlighting its broad therapeutic efficacy across different organs affected by SSHTN. These findings suggest that manipulating macrophage polarization using AVE holds potential for managing SSHTN and its complications. Although our main emphasis was on assessing the influence of AVE on macrophage polarization, it’s essential to recognize its broader effects on BP regulation, oxidative stress, endothelial function, and inflammation. Further research is warranted to elucidate underlying mechanisms, determine long-term effects, and optimize the therapeutic potential of AVE in the clinical setting.

## Clinical perspectives

- In SSHTN mice, pro-inflammatory macrophages accumulate in the kidneys and gonads and are associated with inflammation, increased lymphatic density, and organ damage. However, it was unknown whether shifting macrophage polarization towards an anti-inflammatory state can counteract these effects in renal and gonadal tissues.
- AVE reduced BP, inflammation, inflammation-associated lymphangiogenesis, and organ damage in the kidneys and gonads of SSHTN mice by promoting anti-inflammatory M2 macrophage polarization and concurrently suppressing pro-inflammatory M1 macrophages.
- These results shed light on the therapeutic promise of targeting macrophage polarization to alleviate the effects of SSHTN on inflammation and organ damage. Further exploration is needed to assess the clinical feasibility of this approach in SSHTN management.

## Supplementary Material

Supplementary Figure S1 and Table S1

## Data Availability

All supporting data are included within the article.

## Abbreviations

AVE: AVE0991
BP: blood pressure
DC: dendritic cell
DPBS: Dulbecco’s phosphate-buffered saline
FBS: fetal bovine serum
FENa: fractional excretion of sodium
HTN: hypertension
IFN: interferon
IL: interleukin
L-NAME: nitro-l-arginine methyl ester hydrochloride
LYVE1: lymphatic vessel endothelial hyaluronan receptor 1
NK: natural killer
SBP: systolic blood pressure
SEM: standard error of the mean
SSHTN: salt-sensitive hypertension
Th1: T helper 1
Th17: T helper 17
Th2: T helper 2
Tregs: regulatory T cells
VEGF-C: vascular endothelial growth factor C
VEGF-D: vascular endothelial growth factor D
