# Supplementary material for: Differential changes in end organ immune cells and inflammation in salt-sensitive hypertension: effects of increasing M2 macrophages
Source: Clin Sci (Lond). 2024 Jul 16;138(14):921–40. doi: 10.1042/CS20240699 (PMC11250104; doi:10.1042/CS20240699)
Supplement: Supplementary Figure S1 and Table S1 [file CS-2024-0699_supp.pdf]

DATA SUPPLEMENT

Differential Changes in End Organ Immune Cells and Inflammation in Salt-Sensitive Hypertension: Effects of Increasing M2 Macrophages

Authors and Affiliations

Shobana Navaneethabalakrishnan, Bethany L. Goodlett, Hannah L. Smith, Alyssa Cardenas, Asia Burns, Brett M. Mitchell

Department of Medical Physiology, Texas A&M University College of Medicine, Bryan, TX, U.S.A.

Corresponding Author

Brett M. Mitchell, PhD, ORCID: 0000-0002-2575-8761, brettmitchell@tamu.edu, 8447 Riverside Parkway, Medical Research Education Building II, Bryan, Texas, 77807, USA; Tel: +1 979 436 0751

Competing Interests

The authors declare that there are no competing interests associated with the manuscript.

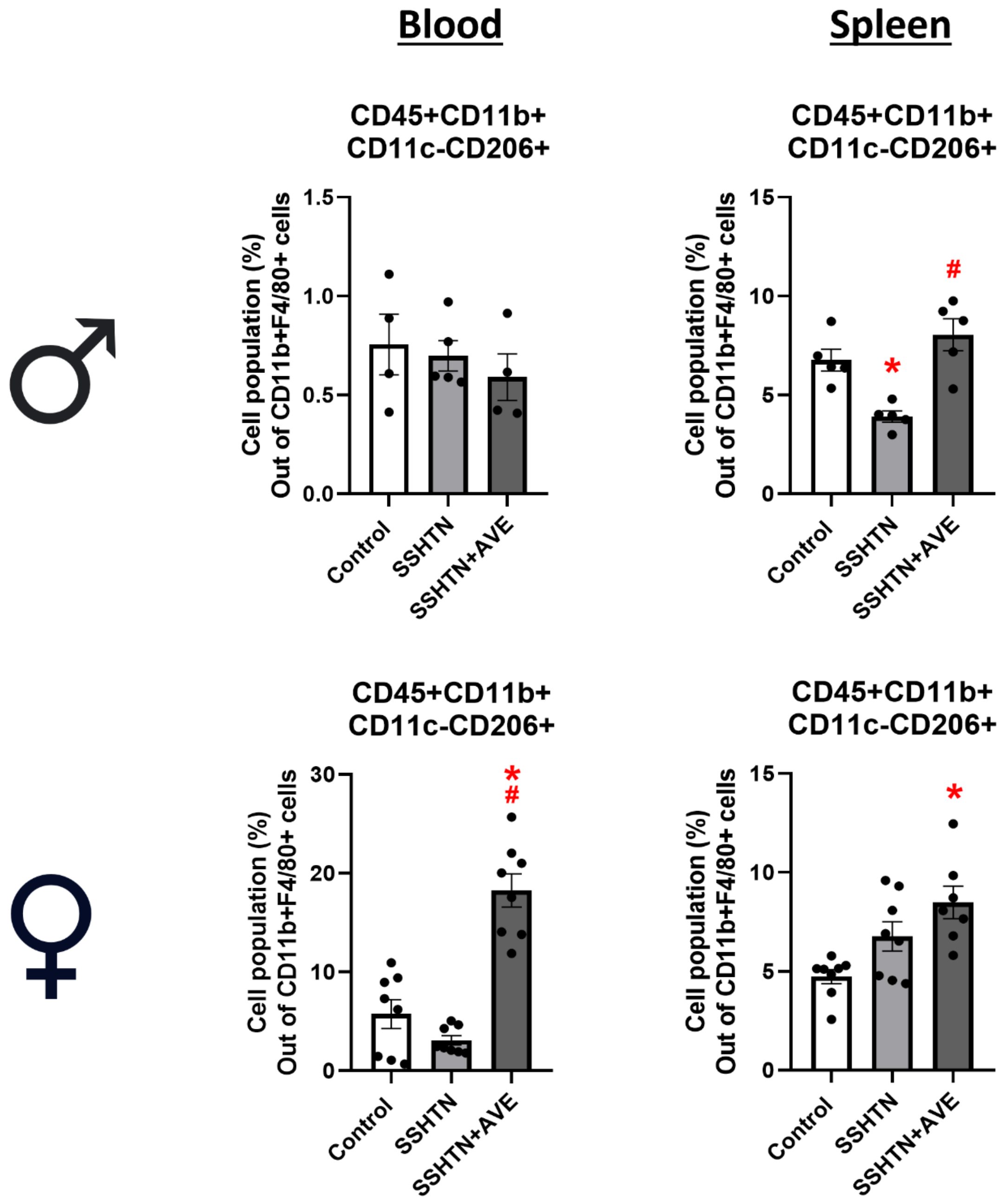

Supplemental Figure 1. AVE0991 treatment mostly increased M2 macrophages in the blood and spleens of male and female mice with SSHTN. Flow cytometric data examining renal populations of M2 macrophages in control, SSHTN, and SSHTN+AVE male and female mice. Each cell population is shown as a percentage of its respective parent population. Results are presented as mean ± SEM and statistical analyses were performed with one-way ANOVA (*n*=4-6 per group). **P*<0.05 vs control mice and #*P*<0.05 vs SSHTN mice.

Online Table I. Flow cytometry antibody panel descriptions for mouse kidneys and gonads.

	**Innate Immune Cells**				
**Fluorochrome**	BV421	FITC	APC	BV480	BV785
**Antigen**	CD45.2	CD11b	CD11c	F4/80	CD206
**Final Conc (µg/mL)**	2 (kidney & ovary) 4 (testis)	5 (kidney & ovary) 10 (testis)	2 (kidney & ovary) 4 (testis)		
**Dilution Factor**	1:100 (kidney & ovary) 1:50 (testis)				
**Clone**	104	M1/70	N418	T45-2342	C068C2
**Manufacturer**	BD	BioLegend	BioLegend	BD	BioLegend

	**Natural Killer Cells**		
**Fluorochrome**	Pacific Blue	BV570	APC-Fire810
**Antigen**	CD3e	CD161	CD19
**Final Conc (µg/mL)**	2 (kidney & ovary) 4 (testis)	1.5 (kidney & ovary) 3 (testis)	2 (kidney & ovary) 4 (testis)
**Dilution Factor**	1:100 (kidney & ovary) 1:50 (testis)		
**Clone**	500A2	PK136	6D5
**Manufacturer**	BD	BioLegend	BioLegend

	**CD4-Lineage T Cells**	
**Fluorochrome**	PE-Vio615	PE-Cy7
**Antigen**	CD4	CD25
**Final Conc (µg/mL)**	1.5 (kidney & ovary) 3 (testis)	2 (kidney & ovary) 4 (testis)
**Dilution Factor**	1:100 (kidney & ovary) 1:50 (testis)	
**Clone**	REA1211	PC61
**Manufacturer**	BioLegend	BioLegend

	**CD4-Lineage T Cells (Intracellular)**				
**Fluorochrome**	BV605	BV711	AlexaFluor 700	PerCP-eFluor710	APC-Cy7
**Antigen**	IFNg	IL4	FoxP3	TNFa	IL17a
**Final Conc (µg/mL)**	2 (kidney & ovary) 4 (testis)				
**Dilution Factor**	1:100 (kidney & ovary) 1:50 (testis)				
**Clone**	XMG1.2	11B11	FJK-16s	MP6-XT22	TC11-18H10.1
**Manufacturer**	BioLegend	BioLegend	eBioscience	eBioscience	BioLegend

	**Viability**
**Fluorochrome**	Zombie UV
**Dilution Factor**	1:200 (all tissues)

**Manufacturer**	BioLegend

Abbreviations: APC = allophycocyanin; BV = brilliant violet; FITC = fluorescein isothiocyanate; PE = phycoerythrin; PerCP-Cy7 = peridinin chlorophyll protein complex cyanine 7
